# Investigating Violence and Control Dyadically in a Help-Seeking Sample from Mozambique

**DOI:** 10.1100/2012/590973

**Published:** 2012-05-15

**Authors:** Nicola Graham-Kevan, Antonio Eugenio Zacarias, Joaquim J. F. Soares

**Affiliations:** ^1^School of Psychology, University of Central Lancashire and School of Psychology, Mid Sweden University, Sundsvall, Sweden; ^2^Faculty of Medicine, Eduardo Mondlane University, Maputo, Mozambique; ^3^Division of Social Medicine, Department of Public Health Sciences, Karolinska Institutet, Stockholm, Sweden; ^4^Department of Public Health Sciences, Institution for Health Sciences, Mid Sweden University, Sundsvall, Sweden

## Abstract

A sample of 1442 women attending a Forensic Healthcare Service provided information on their own and their partners' use of controlling behaviors, partner violence, and sexual abuse, as well as their own experiences of childhood abuse. Using Johnson's typology, the relationships were categorized as Nonviolent, Intimate Terrorism, or Situational Couple Violence. Findings suggest that help-seeking women's experiences of intimate violence may be diverse, with their roles ranging from victim to perpetrator.

## 1. Introduction

Intimate partner violence (IPV) against women is a source of major concern around the world. It is a pervasive problem, poses serious financial threats, has negative effects on intimacy, and causes high rates of morbidity and mortality (e.g., [1, 2]). In Sub-Saharan Africa, the occurrence and negative outcomes of IPV are a serious public health issue that may further aggravate the existing poor socioeconomic and health situation of women. Studies indicate that 5–29% of women are physically assaulted by their male intimates annually and 13–59% during life-time (e.g., [1–10]).

Prevalence rates for sexual assault against women vary by study and time period, during the past 5 years ranging from 0.8 to 4.5%, the past 12 months from 9.1 to 44.4%, and ever abused from 16.5 to 58.6% [1, 2, 4–6, 11, 12] The prevalence of verbal/emotional abuse during the past 12 months and life-time may be as high as 31.3% and 50%, respectively [4, 8]. Different forms of violence often cooccur and at relatively high levels. In a recent report concerning violence in 7 Sub-Saharan African countries, the rates of cooccurring physical/sexual abuse ranged from 6.8 to 24.4% and emotional/physical/sexual from 3.6 to 8.3% [4] (Cameron, Kenya, Malawi, Rwanda, Uganda, Zambia, Zimbabwe.)

Some data indicate that male control is related to increased IPV vulnerability for women [2, 4, 11, 13, 14]. Women's vulnerability to IPV has also been found to be related to experiences of abuse as a child [15, 16]. The literature on violent men, however, suggests that abusive childhoods are also associated with perpetration of IPV (e.g., [17, 18]).

The influence of women's IPV perpetration on their own victimization has not attracted great attention in the Sub-Saharan Africa context, but a few studies suggest that women may initiate and abuse male partners at rates from 0.5 to 27% [2, 7, 8, 13, 14, 19–21] (only studies with women as respondents). In contrast to other countries in Sub-Saharan Africa, there is a lack of data concerning the prevalence and consequences of IPV in Mozambique generally. As far as we know, only one study [3] has explored this topic. The authors found that 11% of women aged 16–60 years (*n* = 1374) had been physically abused by their male partners in the past year. The studies cited previously, however, concentrate on men's perpetration of physical violence towards women. Women's abuse of male partners may not have been sufficiently addressed, particularly the contribution women's perpetration may make to their own victimization.

There is some evidence [14] that women's and men's coercive control may be related to IPV in Sub-Saharan Africa (consistent with data from the West [22]), but most research has investigated this only in relation to male control and IPV [2, 4, 11, 13, 14]. The contribution of women's coercive control in understanding the abuse of men has rarely been investigated, although there are exceptions from Western samples (e.g., [22–24, 24]). Studies using American and European populations have found that women in heterosexual relationships are as likely to use physical and psychological violence as men, and that dominance/control, “multiple” forms of abuse, and repeated abuse are generally equivalent among women and men (e.g., [25–32]). 

There are few studies exploring the relationship between control and women's experiences of IPV in Sub-Saharan Africa, and generally the measurement of control has been weak. An exception is Próspero et al. [33] who used three subscales of the Controlling Behaviors Scale (CBS-R, [23, 24]) to measure men's and women's use of controlling behavior and IPV in a sample of university students in Ghana. They found that men and women reported similar frequencies of controlling behavior victimization and perpetration and that their use of control predicted their use of IPV.

Controversy over the nature of “domestic violence” has been apparent for many years (e.g., [34]). Drawing on data from women's shelters, police, and emergency rooms, feminist researchers have argued that women are the victims of domestic violence [35, 36] with some going so far as to state that “domestic violence” is synonymous with wife abuse [37]. A parallel body of research published over the same time period however challenged this position. Using the Conflict Tactics Scales (CTSs), developed by Straus [38], the National Family Violence Surveys (NFVSs) in 1975 and 1985 found symmetry in the use of physical aggression by men and women against their partners. Although this was initially ignored by researchers and published without comment [39], others were bolder. Steinmetz's [40] analysis of the 1975 NFVS data led to the term “battered husband” being used and a call for more resources to be directed to male victims of partner abuse. Although controversial, the findings of the NFVSs were by no means isolated (see [32]) and so could not be dismissed as anomalies.

Feminist researchers rejected not the findings *per se*, but instead the conclusions drawn from them. Arguing that the context in which IPV is used is crucial to understanding it [41], they questioned the validity of studies using the CTS. Feminist researchers placed men's IPV within a general framework of power and control, with men being reported as using a range of control tactics such as intimidation, threats, isolating the victim from friends and family, and preventing their partner from having access to money [42]. The physical aggression within this context could be seen as an event among many events which all lay on the same continuum of control. Prevalence statistics and frequency analysis, they argued, failed to discriminate between coercive and/or offensive IPV and noncoercive and/or defensive IPV.

Kimmel [43] reviewed this debate and identified several areas of ambiguity including whether the term “gender symmetry” meant that men and women used similar frequencies of IPV, or that men and women used IPV for similar reasons, or whether the consequences of IPV by men and women were equivalent. In regards to frequency, Archer's [25, 44] meta-analyse suggests that men and women use IPV at similar frequencies (at least in Western nations), but that this effect was sensitive to sampling methods. Archer [44] also found that consequences, in terms of injuries, were more likely to be incurred by women than men (62% of those injured were women). This is understandable as men on average have a significant size and strength advantage over women and so, regardless of the motivation, assaults by men are more likely to cause injury or death (the sex ratio for deaths is similar to the ratio for injury with women typically making up approximately two thirds of IPV deaths in the West).

Understanding motivation for IPV use cannot be inferred from frequency data however. As Kimmel [43] rightly states the CTS is a measure of acts of violence and is not designed to measure the circumstances under which such acts occur. Important contextual factors that could be used to infer motivation were identified by him and included who initiated the violence, and what the nature of that violence was, that is, expressive (emotional aggression stemming from losing control) or instrumental (proactive/goal directed aggression used to attain or maintain compliance) violence, as Kimmel states “… motivation for violence matters.” Johnson [45] also argued that the lack of context in act-based studies of IPV obscured important differences between relationships, where IPV was present, and also between men's and women's use of IPV.

In an attempt to explain apparent gender symmetry in IPV in terms of feminist theory Michael Johnson proposed that there were important differences between highly controlling perpetrators and those that, although physically aggressive, were not also controlling. Johnson and ferraro [46] proposed that there were actually qualitatively different types of IPV relationships and that these types were distinct in terms of the harm caused. Like his predecessors [37] Johnson [45, 46] proposed that most people are thinking of *Intimate terrorism* (IT) when they use the term “domestic violence”. He defined IT as a relationship where one partner is highly controlling and physically aggressive whilst the other partner is not controlling (even though they may also be physically aggressive). IT is thought to be most prevalent in criminal justice and female help-seeking samples. This is believed by Johnson to reflect the harmful nature of such relationships.

Because Johnson's relationship types are based on both members of the couple's behavior, he classified the aggressive behavior of the noncontrolling partners in an IT relationship as using *Violent Resistance* (VR). Johnson has written extensively on IT but acknowledges that “… research on the dynamics of VR are surprisingly meagre” and that it is was time to “… give more research attention to the incidence and nature of VR in partner violence” ([46], page 949). In IPV relationships where both partners use physical aggression and high levels of controlling behaviour, Johnson used the term *Mutual Violent Control* (MVC). Although Johnson and Ferraro [46] believed MVC to be rare, they also stated that the dynamic of MVC is similarly little researched and that there is a need to explore the impact that violence from both partners has on relationships.

Johnson [45] and Johnson and ferraro [46] proposed that the most common form of IPV was *Situational Couple Violence* (SCV) which he suggests is the predominant type of IPV that women use and is found in community and student samples. SCV differs from IT in that neither partner is highly controlling of the other (one or both partners use IPV however). Johnson and Leone [47] argue that the most harmful type of relationship is male-perpetrated IT and use their analysis of women's reports (where men's reports of their victimization were omitted even though they were also available) from the NVAWS in support of this assertion. This analysis found that women whose partners were classified as IT experienced the most frequent and injurious IPV, were more symptomatic of posttraumatic stress syndrome, used more painkillers, and missed the most days off work. They were also most likely to leave their husbands to go to a place of safety. Subsequent analysis of the data however has been more equivocal (e.g., [48, 49]). 

The problem with analysis using the NVAS is that it contains victimization data only and so cannot be used to classify relationship dyadically, which is important to enable the behaviors to be understood within the relationship context. Therefore, neither [49], Felson's and Lane's [50], nor [47] analysis can distinguish between IT with a nonviolent partner, IT with a violent but not controlling partner, VR, or two intimate terrorists, MVC. The possible impact of the women's behavior is entirely ignored, even though relationship research is clear in that both partners' behavior need to be explored to understand relationship interactions (e.g., [51, 52]) and that their contribution may be independent of the other partner's behavior [53], even in the case of women's IPV [27]. Consistent with this is research finding that a woman's use of IPV is a significant risk factor for her own victimization (e.g., [24, 50, 54–56]).

Although Johnson does concede that women can be ITs, his writings [45, 46] suggest that physical aggression used by controlling men is more dangerous and hence more likely to be unilateral than other types of IPV relationship [46, 48, 57]. Research supports the contention that as risk of injury a particular behavior incurs increases, so women's involvement in that behavior decreases [58]. Therefore if the relationship dynamic of IT is one of fear inducing violence, then this would be expected to have the effect of inhibiting the woman's aggression. However, Felson and Cares [59] and Felson and Outlaw [48] found that controlling men's IPV was just as likely to be victim precipitated (defined as when the victim was “the first person to use or threaten force during the incident”) as controlling women's.

An explanation of this may be that not all people respond to danger in the same way. Research has found that those subjected to aversive childhood experiences, such as abuse, may respond to perceived threats with aggression themselves. This is likely to be the result of both neurological [60] and psychological [61] factors. The comorbidity of IPV perpetration and abusive childhoods is also consistent with research on convicted male [17, 18, 62, 63] IPV perpetrators. These studies find that “generally violent” men and women are more likely to have childhood abuse histories.

Johnson proposed his typology in 1995 to explain the apparent contradiction between traditional understanding of IPV (e.g., that it was a violent man assaulting a usually passive woman) and the increasing evidence of mutuality and female initiated IPV. Indeed, [64] states that the use of controlling aggression “… is highly gendered, and in heterosexual relationships, is nearly always perpetrated by a man against his female partner” (page 6). Others have argued that the use of controlling violence is more evenly distributed across the sexes and that Johnson's assertion is the result of using biased samples [24, 65–67] and/or inadequate analysis [48]. In nonselected samples controlling aggression does not appear to be more commonly used by men than women ([26, 30, 48]). In analyses of samples of women from crime surveys [47] or women known to authorities as victims of IPV researchers have concluded that men are more likely to be the controlling aggressor than women. The problem with such analyses, however, is that the authors do not control for the self versus other effects.

Research has consistently found that people underreport negative behavior they have engaged in; for example, reporting biases have been investigated in many fields where answers may be evaluated negatively such as medicine (e.g., [68]), nutrition (e.g., [69]), and the media (e.g., [70]), and loss reporting in finance (e.g., Hoffman and Patton, 2002). Within the literature on partner physical aggression, it has been found that self-reports are considerably lower than reports about one's partner (e.g., [71–74]). As such an effect is not found with positively valenced information; it is likely that this is due to socially desirable responding. Reference [75] meta-analysis found that, irrespective of sex, socially desirable responding was related to status, with perpetrators having a stronger relationship than victims.

The present study will therefore use women's reports of their own behavior and reports of their partner's behavior (specifically on frequency of control and the use of one or more acts of physical (not sexual) aggression) to classify the relationship dyadically into either nonviolent, IT, VR, MVC, or SCV. All relationships where IPV is present, either from one or both partners, will then be compared on the frequency of physical and sexual aggression from both partners, on mutuality of IPV and initiation of IPV, on the frequency of women's abuse history, and on the reciprocity of control and IPV.

As the current analysis explores the relationship dynamics of a female help-seeking sample, it is predicted that the most prevalent form of IPV will be IT for the men and VR for the women. It is also expected that female IT and MVC will be the least frequent types of IPV. It is predicted that male IT will be least likely to involve mutual violence and SCV to involve the most. Consistent with Johnson's predictions [76], those men who use controlling aggression will use the most frequent and injurious IPV (with SCV involving the least). Due to the atmosphere of threat believed to exist when a man uses controlling aggression in a relationship, it is expected that although most women will not initiate or respond with aggression, those with childhood histories of child abuse will be more likely to be involved in mutual violence and to initiate violence more frequently than women without such histories. IT and MVC women will be most likely to have childhood abuse histories consistent with the findings on male and female batterers [17, 18, 62, 63].

## 2. Method

### 2.1. Setting and Participants

The participants consisted of 1,500 women aged between 15 and 49 years living in Maputo City, Mozambique (women in these age-spans in Maputo amount to 424,194). The women came in contact with the Forensic Services at the Maputo Central Hospital during one year (consecutive cases) for their experiences of psychological, emotional, sexual, or physical IPV. Classifying these women as victims of IPV is consistent with other research conducted in health settings [77, 78]. The women are a mixture of self-refer, referred by female organizations and police, with the majority being self-refer or referred by female organizations. However, no annotations were made on the exact numbers. Of this sample, 1,442 women accepted an offer to participate in the study and 58 declined (response rate, 96.1%). However, the number of women responding to questions about violence varied between 1,429 and 1,340 depending on the type of violence they had experienced (physical, psychological, sexual, or physical with injury). Therefore, there may be missing data due to questions not being relevant to a respondent.

### 2.2. Measures

#### 2.2.1. Intimate Partner Violence (Physical, Injurious, and Sexual) (IPV)

 IPV was assessed with the CTS2 scales [79]. For the current analysis the 12-item physical aggression scale measured physical aggression towards a partner (e.g., pushed or shoved my partner, beat up my partner). The potential score range for the 12 items is from zero to 300 (12 items with a maximum score of 25 for each item = 300). The 7-item sexual aggression was also used (e.g., made my partner have sex without a condom, used threats to make my partner have oral or anal sex) which had a potential score range of zero to 175. The 6-item injury scale (e.g., had a sprain, had a broken bone from a fight with my partner) was used to measure injurious aggression and had a potential score range of zero to 150. For each item respondents indicated the frequency of occurrence from never, once, twice, 3–5, 6–10, 11–20, or >20 times during the past year. The validity and reliability is good (e.g., [79]). For this study, questions on negotiation were not analysed. Cronbach *α*'s for women as victims were 0.89 for physical assault, 0.73 for sexual coercion, and 0.65 for physical assault with injury. The correspondent *α*'s for aggressors were 0.79, 0.63, and 0.70, respectively.

#### 2.2.2. Controlling Behaviors

 Controlling behaviors were assessed with the CBS-R [24] which has been showed to have good discriminative ability [65]. The CBS-R can be scored to derive five subscores, each of which is a particular type of control tactic, or a total controlling behavior score using all 24 items, which was used in the current analysis (Cronbach for women's self-reports on partners control *α* = 0.93, and for women's reports about their control over partners *α* = 0.91). The respondents used a 5-point response format to indicate how often during the past year with their partners, they had used each behavior, the anchors ranging from 0 (never) to 4 (always) with a possible range of 0–96.

#### 2.2.3. Psychosocial Measures

Who *initiated the physical assault* was assessed in the following way. If you have been physically assaulted by your partner or you physically assaulted your partner, who did it first. Your partner assaulted you first, you assaulted your partner first, or both initiated *Abuse as a child* was assessed with 4 items, one each for physical abuse (e.g., beaten up), psychological abuse (e.g., shouted or yelled at), sexual abuse (e.g., forced to have sex) and injury (e.g., bruised), and chronicity (how often the acts occurred). The acts may have occurred once, twice, 3–5, 6–10, 11–20, or >20 times or never occurred (the scaling was based on CTS2. only studies with women as respondents). The items obtained data about the respondent's exposure to violence before the age of 15 years. Cronbach *α*'s were 0.72 for physical abuse, 0.70 for psychological abuse, 0.68 for sexual abuse, and 0.71 for injury (univariate data are not shown). 

### 2.3. Design and Procedure

Trained female interviewers (medical students at the Faculty of Medicine/nurses at the Forensic Services) carefully informed the women about all details of the research, the degree of their participation, and the way information would be processed. Strong emphasis was put on voluntariness and confidentiality and that nonparticipation would not lead to any negative effects. In the second step, if the women accepted the offer to participate, an interview (on average 1 hour) was performed in a private room by means of a questionnaire. Data processing and their preservation were conducted according to usual anonymous and confidentiality rules rendering public only results from aggregated data. Feedback information on the study will be made available to participants, on request, as aggregate data relationships. The National Ethical Committee at the Ministry of Health of Mozambique approved the study.

## 3. Results

The proportions of women and men using any act of physical (not sexual) aggression towards their partners in the previous 12 months were 38% of women and 44% of men according to the women sampled. The proportions of women and men using any act of psychological aggression towards their partners were 64% of women and 65% of men according to the women sampled. The proportions of women and men using any act of sexual aggression towards their partners were 39% of women and 51% of men according to the reports of the women sampled. It is important to note that all these frequencies were for the past year and are not life-time or relationship rates.

### 3.1. Cut-Off Level for Categorization

Normative sample means for controlling behaviors (self-reports and reports about partners) were used to classify participants and their partners on levels of control. A normative sample [23, 24] was used as the present sample would be expected to contain more frequent controlling behavior use, in the men at least. If this is the case, it would have the effect of under-misclassifying high control [26, 76]. Therefore, those women who reported a controlling behavior frequency of less than two standard deviations higher than the normative sample self-report mean (34.81 for the total CBS-R scale) were classified as not controlling. Those women who reported a controlling behavior frequency of equal to or greater than two standard deviations above the normative sample mean were classified as controlling. The same procedure was used for men, although in this case the normative sample partner reported mean was used (37.38) as reports about partners tend to be higher than self-reports.

Combining the level of control (low or high) with whether *any act of *physical aggression had been used (yes or no), reports were categorized as indicating either nonviolence, noncontrolling violence, or controlling violence. Using women's self-reports, 64% were classified as nonviolent, 26% as using noncontrolling physical aggression, and 9% as using controlling physical aggression. Using the women's reports about her partner, 46% were classified as nonviolent, 32% as using noncontrolling violence, and 22% as using controlling violence.

### 3.2. Classifying Relationships

If neither party used *any act of *physical (not sexual) aggression during the previous year, then the relationship was classified as nonviolent. Dyads where only noncontrolling physical aggression was used (by one or both partners) were labeled Situational Couple Violence (SCV). Dyads where the respondent used no aggression or noncontrolling physical aggression and their partner used controlling physical aggression were labeled as a Victim of Intimate Terrorism (VIT). Dyads where the respondent used controlling physical aggression and their partner used no or only noncontrolling physical aggression were labeled Intimate Terrorism (IT). Dyads where both partners used controlling physical aggression were labeled Mutual Violent Control (MVC). The most common type of relationship was nonviolent (44.7%, *n* = 599), followed by SCV (30.7%, *n* = 412), VIT (15.4%, *n* = 207), MVC (6.3%, *n* = 85), and IT (2.8%, *n* = 37), respectively. Looking at the type relationship in relation to the woman's behavior, just 4.6% of the women's violence is perpetrated against a nonviolent partner (as compared with 36.2% of the men's violence) and 7.9% of the women's violence is IT (compared with 28.8% of the men's violence) (see Table 1).

Those relationships that were nonviolent were excluded from subsequent analyses on violent relationships. Therefore the remaining analyses used only reports where one or both partners used one or more acts of physical aggression towards each other within the past year (*n* = 741).

### 3.3. Relationship Type and Mutuality

With the exception of MVC which is defined in terms of *both* partners using physical aggression, it is possible for one partner to be violent and the other nonviolent. The mutuality of violence use was therefore calculated across the relationship types (see Table 2). The most common profile was mutual violence across all relationships, followed by male partner only. The pattern for SCV is 53.4% mutual and 41.5% man only. For VIT it is 57% mutual and 43% man only, and IT almost exclusively mutual. This suggests that in the present sample there are two dominant patterns in the data: mutual and male-only violence (Table 2).

### 3.4. Relationship Type and Who Hits First

Across relationship types, there were differences in who usually initiated the violence according to the relationship types. The most one-sided was the VIT women who were the least likely to initiate the violence, whereas IT women perpetrators were the most likely (see Table 3). Overall, however, the dominant themes again were mutual initiation which ranged from 25% (MVC) to 43% (IT), and that of man-only initiation which ranged from 38% (IT) to 70% (VIT). Woman-only initiation was the least common, even for those few cases with a woman intimate terrorist, only 19% of the cases involve women only initiation with men-only initiation being double that.

### 3.5. Relationship Type and the Use of Physical Aggression

A between-subjects (SCV, IT, VIT, and MVC) MANOVA compared men's use of acts of physical aggression and injuries sustained by their partners (excluding those men who had used no aggression) (see Table 3). There was a large [80] significant main effect of relationship type on men's acts physical aggression (*F*(3,718) = 331.81, *P* < .0005, Eta^2^.32) and injurious physical aggression (*F*(3,740) = 326.388, *P* < .0005, Eta^2^.31). Scheffe's post hoc tests found that in relationships where there is an IT man or an MVC man, there were significantly more frequent acts of physical aggression used by them than the SCV men. The pattern for injuries is more complex however. Consistent with the results for acts of physical aggression, in relationships where there is an IT man or an MVC man, there were significantly more frequent injuries resulted from their physical aggression than SCV men. However, IT men used significantly less frequent injurious aggression than MVC or men who were victims of women's IT (see Table 4).

A between-subjects (SCV, IT, VIT, and MVC) MANOVA compared women's use of acts of physical aggression and injuries sustained by their partners (excluding those women who had used no aggression). There was a large significant effect of relationship type on women's acts of physical aggression (*F*(3,740) = 76.57, *P* < .0005, Eta^2^.24) and injurious (*F*(3,740) = 40.38, *P* < .0005, Eta^2^.14) aggression. Scheffe's post hoc tests found that IT and MVC women used significantly more of both types of physical aggression compared to SCV or VIT women (see Table 4).

### 3.6. Relationship Type and Frequency of Sexual Aggression Perpetration

A between-subjects (SCV, IT, VIT, and MVC) ANOVA compared men's use of sexual aggression towards their partners (excluding those men who had used no aggression). There was a medium-sized [80] significant main effect of relationship type on men's sexual (*F*(4,740) = 17.95, *P* < .0005, Eta^2^ = .07) aggression. Scheffe's post hoc tests found that sexual aggression was most frequent for controlling violent men (IT and MCV) who used more frequent sexual aggression than noncontrolling violent men (SCV and VIT) (see Table 4).

A between-subjects (SCV, IT, VIT, and MVC) ANOVA compared women's use of sexual aggression towards their partners (excluding those women who had used no aggression). There was a medium-sized significant effect of relationship type on women's use of sexual aggression (*F*(4,740) = 17.54.18, *P* < .0005, Eta^2^ = .07). Scheffe's post hoc tests found that women in MVC relationships used more frequent sexual aggression towards their male partners than did SCV, VIT, or IT women (see Table 4).

### 3.7. Childhood Risk Factors for IPV by Relationship Type

A MANOVA was used to explore exposure to physical, psychological, and sexual aggression during childhood for women in the four relationship types (SCV, IT, VIT and MVC). There was a small [80] significant multivariate effect of relationship type. Univariate analysis found that there was a small but significant effect for exposure to physical aggression (*F*(3,702) = 2.08, *P* = .032, Eta^2^ = .01). Post hoc analysis found that women in MVC relationship reported significantly more incidents of physical aggression victimization (mean = 9.68) than women who were classified as being IT (mean = 2.92).

Univariate analysis found a significant effect for exposure to psychological aggression during childhood for women in the four relationship types (SCV, IT, VIT, and MVC). There was a significant main effect of relationship type (*F*(3,687) = 6.33, *P* < .0005, Eta^2^ = .03). Post hoc analysis found that women in MVC relationship reported significantly more incidents of psychological aggression victimization (mean = 3.02) than women who were classified as being IT (mean = .68), VIT (mean = .61), or SCV (mean = .44).

Univariate analysis found no significant difference in relationship types (*F*(3,687) = .62, *P* = .60) on exposure to sexual aggression.

### 3.8. Reciprocity of Acts of Physical Aggression and Controlling Behaviors between Self and Partner

Reciprocity, as it is used in here, refers to the extent to which the frequency of acts by one partner is related to the frequency of acts by the other partner. Bivariate correlation (Pearson's) was used to explore the reciprocity of acts of physical aggression and controlling behavior (Table 5). The most reciprocal relationship type appears to be MVC where both physical aggression and controlling behaviors are highly correlated. SCV relationships show moderate reciprocity. Where relationships have one controlling and one noncontrolling spouse, there is the least reciprocity, particularly for controlling behaviors. Where there is an IT woman, her acts of physical aggression are moderately related to her partner's and her controlling behavior is unrelated to her partner's. Where the IT is a man however, the relationship between acts of physical aggression is weakest and the relationship between the partner's and respondent's use of control is a weak negative one.

## 4. Discussion

 The present study investigated IPV from a dyadic perspective using help-seeking women's reports of their own, and their partners', behaviors. Using prevalence statistics men and women appeared similar on the use of acts of physical aggression towards a partner. Men and women were more dissimilar on the prevalence of sexual aggression with half of men being reported to have used this, compared to just over a third of women. Half of the men and almost a third of the women used one or more acts of physical aggression within the last 12 months. These figures are similar to those found in a UK sample of men convicted on IPV and their female partners [81]. This suggests that such a pattern may be consistent with the classification of “help-seeking” used in the current study and previous research [77, 78].

The mean levels of control for the classification of *high *controller were similar to the levels reported by women seeking shelter due to IPV from a UK sample that used the same controlling behavior measure [65, 66]. Importantly, this suggests that the cut-off used for the current analysis is appropriate [82]. Most women (74%) who used one or more acts of physical aggression used noncontrolling aggression, whereas over half (59%) of men used controlling aggression. In comparison to previous samples, the figures for women are consistent with Graham-Kevan [26] and Johnson [83]. Prevalence of male controlling aggressors was lower in the current sample than data from a similar sample of help-seeking women in the USA [47]. Although it may be that US men are more likely to be high controllers compared to men from Mozambique, there may also be a cultural explanation. In western nations, there is a presumption of sexual equality and therefore men may need to exert more direct control towards their partners to achieve a similar level of dominance as Mozambique men. Support for this interpretation comes from emerging data from Hong Kong. Here research combining the CBS-R with qualitative interviews with women is finding that a subset (approximately 10%) of the women who reported very low levels of control by their husband appeared to have internalized the ethos of paternal authority to such an extent that their husbands may not need to exert overt control (Tiwari, personal communication, June 2011). This suggests that acceptance of traditional gender roles may be worth exploring in future research.

In the current study, classifying the relationships dyadically revealed that the predominant relationship type was nonviolent. Of those relationships where violence was used, the most prevalent type in this sample was an SCV (56%), followed by relationships with a male intimate terrorist (VIT 28%), male and female intimate terrorists (MVC 11%), and a female intimate terrorist (IT 5%). Within these violent relationships, the majority of relationships were mutually violent, followed by the man only violent, with less than 10% of relationships having a sole female aggressor. There was a higher proportion of nonviolent women in this sample than found in previous studies [31, 84–87], which is to be expected in a sample of help-seeking women. This cannot, however, be used to draw conclusions about the wider gender symmetry/asymmetry debate as the present sample is skewed towards female victims [66].

In order to understand the dynamics of IPV relationships, researchers have been interested in exploring who usually initiates the physical aggression during arguments. Kimmel [43] suggested that information regarding initiation can allow inferences about the motivation to use IPV to be made. Research has found that women typically initiate IPV more frequently than men (e.g., [88–92]. In the present help-seeking sample a little over one in ten women (14%) reported that they hit first, whereas over half (57%) of the women reported that their partners hit first. Indeed across all relationship types, women reported that their partner was more likely to initiate than they were. A third of the time initiation appeared to be mutual. As expected, the rate of women's initiation varied by the relationship type with intimate terrorist women being most likely to report hitting first, and SCV and female victims of IT the least. The man was most likely to hit out first when he was an intimate terrorist and least likely when he was a victim of a female intimate terrorist. That the victims of IT partners (both male and female victims) were least likely to initiate, an attack is consistent with findings from other help seeking populations [93]. These figures cannot be compared to previous analysis of Johnson's typology as this initiation has not previously been explored. Consistent with Kimmel [43], initiation does appear to be a contextual variable that may help to differentiate between different types of IPV relationship. The predominance of male-initiated IPV in the current sample suggests that their partners may be acting in self-defence. Research has found that women reciprocate aggression for a variety of reasons even in clinical populations however, and therefore further research is needed before firm conclusions can be drawn. Although women do describe their aggression as sometimes being self-defensive, they also use descriptions that are more consistent with retaliation, retribution, and vigilantism however [94–96]. These studies suggest that women's partner violence cannot be assumed to be purely defensive, even in samples of highly victimized women. Future research should explore reciprocal aggression in terms of cognitive scripts [97], personality (e.g., [27, 98]), as well as the impact reciprocating aggression on the incident and the relationship. In addition, it is necessary to explore the impact of using *self* versus *reports about others* on reported rates. The literature on reporting bias on negatively valenced behaviors would suggest that using one person to report on their own and their partner's negative behavior (such as starting a physical fight) would create bias that would need to be controlled for. This was not possible in the current study as the authors are unaware of any published research that has investigated this.

 Comparing the frequency of noninjurious and injurious physical aggression and sexual aggression across relationship types revealed a pattern whereby intimate terrorists (both men and women) are the most aggressive individuals. This is consistent with Johnson's predictions but is also consistent with those who have argued that Johnson's typology is an artefact of the linear relationship between control and IPV [23, 24]. The pattern of reciprocity in the current study does provide some support for Johnson's interpretation, with relationship types showing differing patterns of reciprocity. These associations suggest that in relationships where both partners are highly controlling and aggressive there is the strongest evidence for reciprocal behavior. This suggests that in such relationships both parties would benefit from engaging in interventions aimed at changing their dysfunctional behavior [99]. The association between the respondent's and their partner's aggression and control in relationships where partners used noncontrolling aggression was weaker but still suggested reciprocity. It may be that the frequency of control and aggression *by both partners *have an additive effect. This is consistent with previous research that has studied mutually violent heterosexual (e.g., [100]) and homosexual (e.g., [101]) couples. Where partners are mismatched in terms of controlling behaviour, a different pattern arises. When there is a male intimate terrorist, his aggression is only weakly related to his partners' aggression. His use of control, however, is negatively related to his partners, suggesting that the more control he uses, the less his partner uses. With a female intimate terrorist, a similar relationship is found for aggression, but for controlling behaviour, the women's control is unrelated to her partner's. This pattern has not been reported before. It may be a pattern found in help-seeking women's samples only or it may be that there are sex-differences in reciprocity in intimate terrorist relationships.

There was a trend in the mean scores for the three types of aggression in the current sample that suggested that MVC couples have the highest frequency of aggressive behaviour of all IPV relationship types. The existence of MVC couples was suggested as early as 1971 [102]. In these couples, the analysis suggests that as each partners' control and aggression increase, so does the other's abusive behavior. As men are generally physically stronger than women, women in particular would be at an increased risk of serious injury in such relationships compared to nonviolent women living with an IT man.

 It may be that Johnson is correct in identifying IT as the most damaging form of IPV *psychologically*, but that MCV is actually the most *physically* damaging. Such couples may be less likely to identify IPV as problematic and hence less likely to seek help [103]. The relationship literature suggests an explanation as to why this may be so. Gonzaga et al. [53] suggest that similarity in behaviors between partners is validating because each perceives that their emotions are shared with their partner. They suggest that the benefits of emotional similarity are context-free and independent of levels of emotional experience. They use the example of a dyad where both partners have similarly high levels of anger during conflict, positing that this couple would have relational advantages over another couple where their anger levels were dissimilar. The more similar couple would understand each other's emotional experiences better, be able to coordinate their conflict responses, and feel validation from their partners due to shared emotion.

Equally it may be that assortative partnering [22] results in men and women who share similar risk factors for IPV (e.g., antisocial behavior in childhood and mental health problems, [104]) forming relationships. The latter explanation is consistent with the finding in the present sample that women in the MVC group appeared to have the highest levels of risk (childhood abuse). Childhood abuse is a known risk factor for violent men, particularly those who are found in batterer programmes [18]. Either (or both) explanations may explain why MVC (men's and women's) was more common in Johnson's [82] court sample than his women's shelter sample: court samples are the result of the criminal justice system identifying a problem whereas women's shelters are the result of the women identifying a problem. This would suggest that one would find the most asymmetrical relationships in help-seeking women.

### 4.1. Limitations

This study used only one partner to provide frequencies for both her own, and her partner's, physical aggression and controlling behavior. The literature on self versus partner reports suggests that respondents tend to respond in a socially desirable manner by reporting that their partners are more aggressive than they are [71]. In a research that asks couples to report on each other's aggression, findings suggest that perpetrator and victim reports are frequently incongruent (e.g., [105]), with women reporting more aggression by their partner than their male partner reports for himself [106–108]. Another research has found that both men and women report more aggression from partners compared to toward partners [99, 109]. This suggests that wherever possible both partners should provide information on aggression-related variables [74].

### 4.2. Conclusion

The present study has moved beyond classifying violent people to exploring the relationship in terms of both members' use of control and aggression. This analysis suggests that mutuality is a risk factor for more frequent and injurious violence, and hence the behavior of both parties is important to the understanding of IPV. It is important to emphasize that the current analysis does not inform on who is responsible for the IPV. It does however suggest that for those wishing to understand and/or intervene in IPV relationships, it is necessary to explore *both* partners' conflict related behaviors. Treating one person's problematic behavior but ignoring the others may considerably decrease treatment efficacy [110].

## Figures and Tables

**Table 1:** Violence types by gender.

	Women (*n* = 1341)			Men (*n* = 1341)	
Violent	35.9%	(482)	Violent	53.7%	(720)
SCV/SCV*	45.6%	(220)	SCV/SCV*	30.6%	(220)
SCV/NV	4.4%	(21)	SCV/NV	23.8%	(171)
VR/IT	24.5%	(118)	VR/IT	5.1%	(37)
MVC/MVC	17.6%	(85)	MVC/MVC	11.8%	(85)
IT/VR	7.7%	(37)	IT/VR	16.4%	(118)
IT/NV	0.2%	(1)	IT/NV	12.4%	(89)
	100%	(482)		100%	(720)
Nonviolent	64.1%	(859)	Nonviolent	46.3%	(621)
NV/NV	69.7%	(599)	NV/NV	96.5%	(599)
NV/SCV	19.9%	(171)	NV/SCV	3.4%	(21)
NV/IT	10.4%	(89)	NV/IT	0.2%	(1)
	100%	(859)		100%	(621)

*The first term refers to the primary individual, the second to his/her partner. For example, 45.6% of the violent women were involved in SCV with SCV partners, 30.6% of the violent men were involved in SCV with SCV partners, 4.4% of the violent women were involved in SCV with NV partners, and 23.8% of the violent men were involved in SCV with NV partners.

**Table 2:** Mutuality of violence by relationship category (intimate terrorism and situational couple violence only *n* = 741).

Type	Woman only	Man only	Both	Row total
SCV	5.1% (21)	41.5% (171)	53.4% (220)	100% (412)
VIT	—	43% (89)	57% (118)	100% (207)
MVC	—	—	100% (85)	100% (85)
IT	2.8% (1)	—	97.3% (37)	100% (37)

Type: relationship type. Classified as self only if participant had used any physical aggression in the past year and their partner had not used any. Classified as both if participant and their partner had both used physical aggression in the last year.

**Table 3:** Prevalence (and numbers) for violence initiation by relationship type.

		Who hit first			Total
		Woman	Man	Both	
Relationship type	SCV	12.5%	51.7%	35.8%	100%
		(51)	(211)	(146)	(408)
	VIT	13.0%	69.6%	17.4%	100%
		(27)	(144)	(36)	(207)
	MVC	17.6%	58.8%	23.5%	100%
		15	50	20	85
	IT	18.9%	37.8%	43.2%	100%
		(7)	(14)	(16)	(37)

Total		100	419	218	737
		13.6%	56.9%	29.6%	100%

**Table 4:** Women's and men's mean (and standard deviations) aggression by relationship type.

	Women's aggression (*n* = 741)				Men's aggression (*n* = 719)			
Type	SCV	VIT	MVC	IT	SCV	VIT	MVC	IT
Control	13.97	16.68	46.86	43.14	19.95	33.76	51.69	55.57
	(9.28)	(11.00)	(10.52)	(7.29)	(10.56)	(4.33)	(12.86)	(14.20)
PA	10.15	12.21	50.36	48.05	30.30	70.17	71.29	49.86
	(21.87)	(22.74)	(44.09)	(28.31)	(46.38)	(69.42)	(59.12)	(32.16)
Injuries	3.93	6.73	20.62	20.16	6.22	13.01	24.93	23.44
	(11.54)	(14.01)	(23.85)	(20.29)	(13.59)	(20.17)	(23.61)	(17.47)
Sexual	8.56	8.98	22.27	13.00	18.79	34.72	39.93	15.19
	(15.05)	(14.74)	(23.03)	(14.39)	(27.38)	(39.65)	(39.47)	(16.94)

PA denotes acts of physical aggression, irrespective of injuries. Injuries denotes the frequency of injuries sustained as a result of the acts of physical aggression. Control: 0–96 range.

**Table 5:** Associations between self (women's) and partner (men's) use of acts physical aggression and controlling behaviors by relationship type.

	Relationship type			
	SCV	IT	VIT	MVC
Physical aggression	.52**	.46**	.36**	.69**
Controlling behavior	.56**	.16	−.36**	.73**
